# Which screening tool performs best in identifying malnutrition risk among hospitalized older adults with cardiovascular disease? A diagnostic accuracy study comparing six different screening tools with GLIM criteria

**DOI:** 10.1007/s41999-025-01187-y

**Published:** 2025-03-27

**Authors:** Kevser Tarı Selcuk, Sedat Arslan, Ayça Aydın, Duygu Durmaz

**Affiliations:** 1 Department of Nutrition and Dietetics, Faculty of Health Science, Bandirma Onyedi Eylul University, Balikesir, Turkey; 2 Department of Nutrition and Dietetics, Faculty of Health Science, Istanbul Okan University, Istanbul, Turkey; 3 Department of Cardiovascular Surgery, Faculty of Medicine, Bandirma Onyedi Eylul University, Balikesir, Turkey

**Keywords:** Malnutrition, GLIM criteria, Nutritional screening tools, Cardiovascular disease

## Abstract

**Purpose:**

This study aimed to compare the performance of six different screening tools with the GLIM criteria in identifying malnutrition risk among older adults with cardiovascular diseases.

**Methods:**

In this diagnostic accuracy study, data from 669 patients aged 65 and older, hospitalized at Bandırma Training and Research Hospital diagnosed with cardiovascular disease, were evaluated. The data were obtained using a questionnaire that included a Demographic Information Form, Global Leadership Initiative on Malnutrition (GLIM) Criteria, Malnutrition Screening Tool (MST), Malnutrition Universal Screening Tool (MUST), Short Nutritional Assessment Questionnaire (SNAQ), Mini Nutritional Assessment-Short Form (MNA-SF), Nutritional Risk Screening-2002 (NRS-2002), and Graz Malnutrition Screening (GMS) tool. GLIM criteria were used as gold standard and six screening tools were used as index tests. Receiver Operating Characteristic (ROC) curve analysis was conducted to calculate the Area Under the Curve (AUC), and the sensitivity, specificity, accuracy, and Cohen's Kappa (κ) coefficient of the index tests were assessed.

**Results:**

The mean age of the patients was 75.5 ± 7.7 years, 55.2% were female. According to the GLIM criteria, the prevalence of malnutrition was calculated as 22.0%. The AUC calculated by ROC indicated that MST (AUC: 0.905) had excellent predictive value, while MUST (AUC: 0.874), SNAQ (AUC: 0.851), MNA-SF (AUC: 0.842), and GMS (AUC: 0.820) demonstrated good predictive value. Among the screening tools, GMS had the highest sensitivity (92.5%), whereas MNA-SF exhibited the highest specificity (91.6%). MNA-SF also demonstrated the highest agreement with the GLIM criteria (Cohen's κ: 66.8) and the highest accuracy (88.3%).

**Conclusion:**

This study found that the MNA-SF tool outperformed other comprehensive screening tools when evaluating malnutrition risk in light of the GLIM criteria.

## Key summary points

**Aim:**

This study aimed to compare the performance of six different screening tools with GLIM criteria in detecting the risk of malnutrition in hospitalized older adults with cardiovascular disease.

**Findings:**

MNA-SF exhibited the highest specificity (91.6%), the highest agreement with the GLIM criteria (Cohen’s κ: 66.8), and the highest accuracy (88.3%).

**Message:**

MNA-SF has shown the potential to be a preferred screening tool for detecting malnutrition risk in older adults with cardiovascular disease.

## Introduction

The European Society for Clinical Nutrition and Metabolism [ESPEN] has endorsed the definition of malnutrition as “a condition resulting from reduced intake or lack of intake of nutrients that leads to altered body composition (reduced lean mass) and body cell mass, resulting in reduced physical and mental functioning and impaired clinical outcomes from diseases” [1, 2]. Malnutrition can negatively affect clinical outcomes, quality of life, length of hospital stay, risk of readmission and mortality rates, and increased healthcare costs [3–5]. Malnutrition is common in older adults affected by cardiovascular diseases in hospital practice due to conditions such as heart failure, anorexia, pre-investigate 'nil by mouth’ and malnutrition increases the risk of mortality in these patients by 2–10 times [6, 7]. Furthermore, in older adults with cardiovascular disease, increased basal metabolic rate and decreased energy expenditure due to cardiac load, respiratory load, and increased peripheral oxygen consumption may initiate the catabolic process leading to cardiac cachexia. Cardiac cachexia is defined as “the involuntary loss of 6% or more of the patient's dry weight within 6 months” [8, 9]. Cardiac cachexia, which affects approximately 5–15% of patients with cardiovascular disease, is a serious complication associated with a poor prognosis [10]. Therefore, prevention of malnutrition is critical to protect individuals with cardisovascular disease from cardiac cachexia [11].

Although screening for malnutrition during hospital admission is strongly recommended in patients with cardiovascular disease, malnutrition often goes unrecognized and therefore untreated in daily practice [4, 12, 13]. However, assessing malnutrition as a major modifiable risk factor for poor clinical outcomes in this patient group can help select the best diagnosis and treatment for each patient, reduce hospital costs, and guide tailored interventions for secondary prevention, including diet, nutritional supplements, and exercise programs [3–5].

Although malnutrition is common in older adults with cardiovascular disease with a prevalence of up to 56% and is associated with poor prognosis, there are no standardized diagnostic criteria [3, 14–17]. This has led to the development of various screening tools with different validity values [3]. Global Leadership Initiative on Malnutrition (GLIM) criteria were recently proposed to build a global consensus on diagnostic criteria for malnutrition and have been endorsed by several international nutrition and dietetics societies (The American Society for Parenteral and Enteral Nutrition, ESPEN, The Latin-American Federation for Parenteral and Enteral Nutrition, The Parenteral and Enteral Nutrition Society of Asia). The first step in the GLIM criteria involves screening for malnutrition using any validated screening tool. This initial step is crucial for identifying individuals at risk, before proceeding with further assessments based on phenotypic and etiological criteria. For patients considered at risk of malnutrition based on nutritional screening, the GLIM criteria recommend a nutritional assessment that includes phenotypic criteria (involuntary body weight loss, low Body Mass Index (BMI), and reduced muscle mass) as well as etiologic criteria (reduced food intake or malabsorptive disorder, disease burden or inflammation). At least one phenotypic criterion and one etiologic criterion should be present to diagnose malnutrition. In addition, the GLIM criteria rate the severity of malnutrition as moderate or severe [18]. Recently, malnutrition defined according to GLIM criteria has been reported to be a predictor of both low physical function and mortality in hospitalized patients with cardiovascular disease [19]. Furthermore, nutritional care is recognized as a fundamental human right, emphasizing the need for equitable access to adequate nutrition for all individuals. As stated in the Vienna Declaration ensuring proper nutrition is essential for maintaining human dignity and health. By adhering to the GLIM criteria, healthcare providers can offer better targeted interventions that respect the fundamental right to nutrition [20]. On the other hand, there are many validated screening tools in the literature, such as Mini Nutritional Assessment-Short Form (MNA-SF), Malnutrition Universal Screening Tool (MUST), Nutritional Risk Screening-2002 (NRS-2002), Graz Malnutrition Screening (GMS) and Malnutrition Screening Tool (MST), Short Nutritional Appetite Questionnaire (SNAQ), which assess malnutrition risk or malnutrition in the older adults and/or patients with cardiovascular disease [6, 13]. An ideal malnutrition screening tool should be practical, noninvasive, inexpensive, provide rapid results, and have high validity, reliability, and concordance to accurately identify risk, obtain consistent results, and minimize subjective bias [3, 6]. The performance of these screening tools used in different populations is uncertain in hospitalized older adults with cardiovascular disease who are particularly vulnerable to malnutrition due to the combined effects of cardiovascular disease, metabolic changes associated with aging, and factors related to hospitalization.

Recent studies have aimed to harmonize and standardize malnutrition screening tools across healthcare settings [21]. A systematic review recommended the MST, NRS-2002, and MNA-SF as valid tools, with MST being the most widely applicable. Based on this evidence, the Norwegian Directorate of Health issued a national guideline recommending MST as the universal screening tool for all adults [22]. However, these studies did not investigate how different screening tools perform in disease-specific populations, particularly in older adults with cardiovascular diseases. Our study aims to fill this gap by assessing the diagnostic accuracy of six different screening tools in hospitalized older adults with cardiovascular diseases, using GLIM criteria as the reference standard.

## Methods

### Study type and sample

**Fig. 1:**
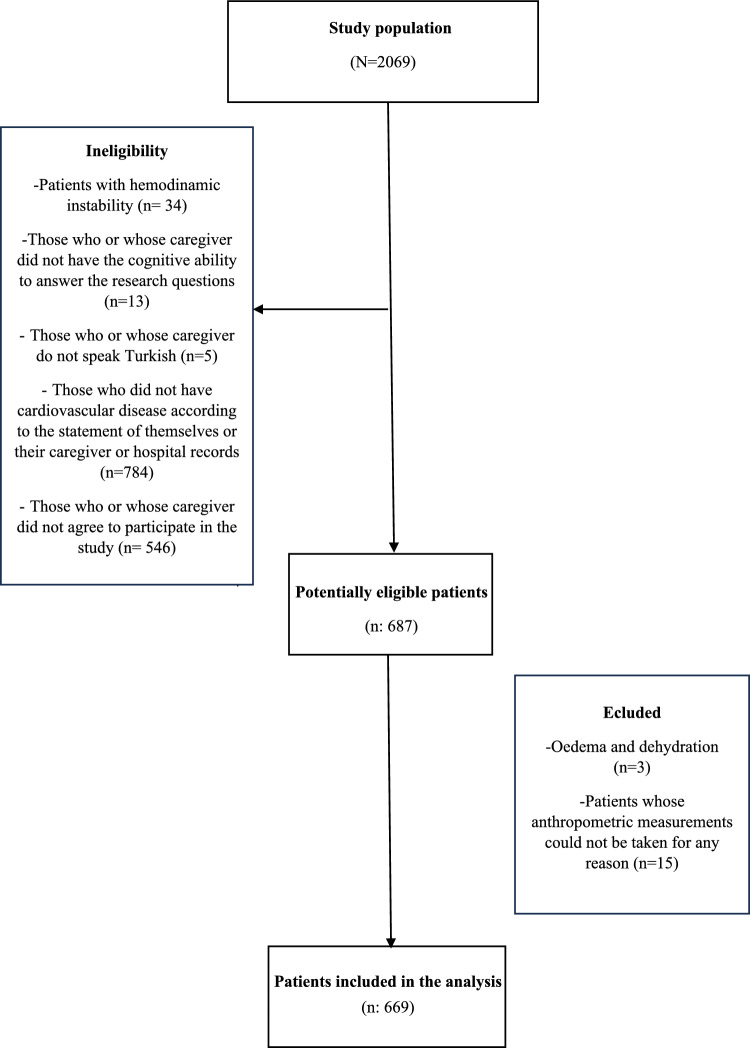
Flowchart of participant inclusion in the study

This study is a diagnostic accuracy study. The minimum sample size to be reached in the study was calculated as 362 people with a 5% margin of error and 95% confidence interval, taking into account the prevalence of malnutrition risk calculated in the study by Özyiğit et al. (2018) in the OpenEpi 3.01 program (38%), and it was planned to include at least 471 patients in the study by taking 30% reserve [23]. Cardiovascular disease was defined according to the American Heart Association (AHA) criteria and included Coronary Artery Disease (CAD), Heart Failure (HF), Atrial Fibrillation (AF), valvular heart disease, Peripheral Artery Disease (PAD), and cerebrovascular disease [24]. The population of this study consists of 2069 patients aged 65 and over who were hospitalized in the internal medicine and surgical clinics of XX Training and Research Hospital between July 1, 2024, and September 30, 2024. Patients with hemodynamic instability (*n* = 34), those who or whose caregiver did not have the cognitive ability to answer the research questions (*n* = 13), those who or whose caregiver do not speak Turkish (*n* = 5), those who did not have cardiovascular disease according to the statement of themselves or their caregiver or hospital records (*n* = 784), and those who or whose caregiver did not agree to participate in the study (*n* = 546) were not included in the study. The remaining 687 patients have been considered potentially eligible. Among the 687 patients included in the study, patients with edema and dehydration (*n* = 3) and patients whose anthropometric measurements could not be taken for any reason (*n* = 15) were excluded and the data of 669 patients were evaluated within the scope of the study (Fig. 1).

### Data collection tools

The data of the study were collected using a questionnaire including the Descriptive Information Form, GLIM Criteria, Malnutrition Screening Tool (MST), Malnutrition Universal Screening Tool (MUST), Short Nutritional Assessment Questionnaire (SNAQ), Mini Nutritional Assessment-Short Form (MNA-SF), Nutritional Risk Screening-2002 (NRS-2002), Graz Malnutrition Screening Tool (GMS).

#### Descriptive information form

This form was prepared by the researchers and consisted of questions about age, gender, marital status, education level, smoking status, alcohol consumption status, presence of comorbidity, surgical operation in the last 3 months, presence of food allergy, diet, disease-specific diet, duration of hospitalization, etc.

#### Global leadership ınitiative on malnutrition (GLIM) criteria

Criteria recommended for use in diagnosing malnutrition, which mainly includes phenotypic criteria such as involuntary body weight loss, low BMI, and reduced muscle mass, and etiologic criteria such as reduced food intake or malabsorptive disorder, disease burden or inflammation. At least one phenotypic criterion and one etiologic criterion must be present to diagnose malnutrition [18, 25]. In this study, phenotypic and aetiological criteria were evaluated as follows:

#### Involuntary body weight loss

According to the statement of the patient or his/her caregiver, a loss of more than 5% of the patient's body weight in the last 6 months or a loss of > 10% in more than 6 months was accepted as involuntary body weight loss [18].

#### Low body mass ındex (BMI)

A BMI value calculated based on body weight and height measurements of < 20 kg/m2 in people under 70 years of age and < 22 kg/m2 in people aged 70 years and over was considered as low BMI [18].

#### Reduced muscle mass

Reduced muscle mass was assessed using Mid-Upper Arm Circumference (MUAC), which is a widely accepted anthropometric indicator of muscle mass in older adults. Mid-Upper Arm Circumference (MUAC) of less than 23 cm in men and 22 cm in women was considered as reduced muscle mass [18, 26].

#### Reduced food intake or malabsorptive disorder

Reduction of energy requirement by ≤ 50% for more than 1 week or by any percentage for more than 2 weeks or any chronic gastrointestinal condition that adversely impacts food assimilation or absorption is defined as reduced food intake or malabsorptive disorder [18].

#### Disease burden or inflammation

Inflammation was evaluated according to the guideline for the evaluation of etiological criteria for inflammation. In this study, inflammation was assessed according to the presence of acute or chronic diseases, infections or injuries associated with inflammation and CRP levels as specified in the guidelines [25]. A CRP level above 10 mg/L is considered inflammation [18, 25].

In this study, GLIM criteria were used as the gold standard, and screening tools such as MST, MUST, SNAQ, MNA-SF, NRS-2002, and GMS were used as index tests.

#### Malnutrition screening tool (MST)

It is a screening tool recommended for hospitalized patients, consisting of two parameters related to malnutrition due to recent involuntary weight loss and loss of appetite. A score of 0–5 is obtained from this screening tool, and if the screening result is ≥ 2 points, the patient is considered to be at risk of malnutrition [27, 28].

#### Malnutrition universal screening tool (MUST)

Although this screening tool was developed to detect the risk of malnutrition in the community due to its rapid results and practicality in terms of use, it is now frequently used in hospitals and nursing homes [29]. Scoring is based on BMI, weight loss in the last 3–6 months, and acute illness score. A score of 0 on the screening tool indicates low risk, 1 indicates moderate risk and ≥ 2 indicates high risk [30].

#### Short nutrition assessment questionnaire (SNAQ)

This questionnaire was developed by Dutch researchers in 2005 and adapted into Turkish by İlhan et al. (2018) for use in patients aged 65 years and older. The questionnaire consists of three questions inquiring about the individual's involuntary body weight loss in the last 3 and 6 months, appetite status in the last month, and use of nutritional support or feeding tube in the last month [20, 21]. In the questionnaire, which is evaluated over 7 points in total, a total score of ≤ 1 indicates no risk of malnutrition, 2 indicates a moderate risk of malnutrition, and ≥ 3 indicates “severe malnutrition and the need for nutritional intervention” [32].

#### Mini nutritional assessment-short form (MNA-SF)

The form developed by Rubenstein et al. [2001] and adapted into Turkish by Sarıkaya et al. [2015] is used in screening for nutritional deficiency in older adults. The form, which consists of a total of 6 items, is scored by taking into account changes in appetite, decreased food intake in the last 3 months, weight loss in the last 3 months, mobility, presence of psychological stress or acute illness, presence of neuropsychological problems and BMI. According to the score obtained from the form, patients are grouped as normally fed [12–14 points], at-risk [8–11 points], or marked malnutrition [0–7 points] [33, 34].

#### Nutritional risk screening-2002 (NRS-2002)

This screening tool was developed by Kondrup et al. [2002] and adapted into Turkish by Bolayır et al. (2019) for use in hospitalized patients. The NRS-2002 consists of two parts: pre-screening and main screening. The pre-screening consists of verbal questions about the individual's BMI value, body weight loss, changes in food consumption, and general condition and is answered as “yes” or “no”. If one of these questions is answered with “yes”, the actual screening continues. If all questions are answered “no”, the patient is rescreened every week. The main screening consists of two parts: “nutritional status” and “disease severity”. Depending on the individual's condition, both sections are scored as “no problem”, “mild”, “moderate” and “severe”. Each section is scored between 0 and 3. In patients aged 70 years and over, 1 point is added to the score due to age. Patients with a total score ≥ 3 are considered to be at risk of malnutrition [35, 36].

#### Graz malnutrition screening (GMS)

This screening tool was developed to screen the risk of malnutrition in the hospital setting regardless of gender, age, and diagnosis and was adapted into Turkish by Sahin and Tek [2022] for use in hospitalized patients [37]. In the first part of the screening tool, the body weight loss of the individual in the last 3 months is evaluated as a percentage and a score between 0 and 2 is made. In the second part, BMI values of individuals under the age of 65 and over the age of 65 are scored between 0 and 2. In the third section, loss of appetite, nausea/vomiting/diarrhea, and chewing-swallowing problems that may cause changes in the individual's food consumption in the last month are questioned and scored between 0 and 3. In the fourth section, the severity of the disease of the individual is questioned and a score between 0 and 2 is made together with the physician. Finally, in patients aged 65 years and over, 1 point is added to the score due to age. If the total score is ≥ 3, the person is considered to be at risk of malnutrition [38].

### Data collection method

Within the scope of the study, the participants were informed about the purpose and scope of the study, and the older adults or their caregivers who agreed to participate in the study and who decided to be included in the study according to the inclusion and exclusion criteria were asked to sign the informed consent form. The questionnaire, which included the Descriptive Information Form, GLIM Criteria, MST, MUST, SNAQ, MNA-SF, NRS-2002, and GMS screening tools, was administered by face-to-face interview method within 48 h following hospital admission to older adults or their caregivers who signed the informed consent form by the researchers. Researchers who collected the data were trained in the use of GLIM criteria before collecting the data. The training included a review of the GLIM guidelines [18, 25]. Then, body weight, height, calf, and upper mid-arm circumferences were measured by the researchers using standardized techniques [29]. The body weight of the older adults was measured by the researchers using a portable ‘Seca’ brand digital weighing scale that can measure accurately up to 100 g with light clothing and shoes removed. Height was measured in centimeters (cm) using a portable height gauge with a capacity of 14–200 cm and 1 mm intervals in centimeters (cm) while the individual was standing in an upright position in the Frankfurt plane (the ear canal and the lower border of the orbit were aligned and the gaze was parallel to the ground). Body Mass Index (BMI) was calculated by dividing body weight (in kilograms) by the square of height (in meters). Mid-Upper Arm Circumference (MUAC) was measured by bending the arm 90° at the elbow while the person was standing, marking the midpoint between the acromial process at the shoulder and the olecranon process at the elbow, and measuring the circumference passing through that point with a non-flexible tape measure without applying pressure on the tissue. Calf circumference was determined by bending the leg 90° with the person lying supine and measuring the circumference of the widest point with a tape measure [29].

### Data analysis

Data were evaluated in the SPSS 23.0 statistical package program. Descriptive statistics (number, percentage, mean, and standard deviation) were calculated in data analysis. The conformity of the distribution to the normal distribution was evaluated with kurtosis and skewness coefficients and values in the range of ( – 1; + 1) were accepted as conforming to the normal distribution. Mann Whitney *U* test was used to compare the mean anthropometric measurements between patients with and without malnutrition according to GLIM criteria. Pearson chi-square test was used to analyze whether there was a difference between patients with and without malnutrition in terms of descriptive characteristics. GLIM criteria were considered the gold standard for the evaluation of sensitivity and specificity. ROC curves were plotted for the six different screening tools compared and the area under the curve (AUC) was calculated to assess the ability of the screening tools to accurately discriminate patients at risk of malnutrition according to GLIM criteria. Area Under the Curve (AUC) values for each ROC curve were interpreted as: moderate (0.70–0.80), good (0.80–0.90), and excellent (> 0.90) [39]. The sensitivity, specificity, Positive Likelihood Ratio (+ LR) and Negative Likelihood Ratio ( – LR), Positive Predictive Value (PPV), Negative Predictive Value (NPV), accuracy, and Cohen's Kappa (*κ*) coefficient were calculated to measure the agreement between all screening tools for classifying malnutrition risk. The Shrout classification was used to interpret κ values as follows: 0–0.10 virtually no agreement, 0.11–0.40 slight agreement, 0.41–0.60 fair agreement, 0.61–0.80 moderate agreement, and 0.81–1.00 substantial agreement [40]. The significance level of statistical tests was accepted as *p* < 0.05.

### Ethical approval

The study was approved by the XX University Health Sciences Non-Interventional Research Ethics Committee (Decision date and number: June 05, 2024/2024-108).

### Informed consent

Informed consent was obtained from all participants.

## Results

The mean age of the patients was 75.5 ± 7.7 years, 55.2% were female, 74.1% were married, and 83.1% had primary school education or less. The proportions of patients who smoked cigarettes and consumed alcohol with varying frequency and amounts were 6.1% and 1.0%, respectively. 60.2% of the patients had at least one comorbid disease. 20.8% of the patients had undergone surgery in the last 3 months. 3.3% of the patients had food allergies, and 95.1% were fed orally. The proportion of patients who followed a disease-specific diet was 45.0%. 81.5% of the patients were hospitalized in internal units and the mean duration of hospitalization was 4.2 ± 8.1 days. The mean body weight, height, BMI, MUAC, and calf circumference were 72.9 ± 14.7 kg, 163.4 ± 8.5 cm, 27.3 ± 5.2 kg/m^2^, 28.5 ± 4.1 cm and 33.9 ± 4.6 cm, respectively. The rates of patients with and without malnutrition according to GLIM criteria were significantly different between those with comorbidities, those who followed a disease-specific diet, and those who were feed orally and enteral nutrition (*p* < 0.05). While the mean age and length of hospital stay of patients with malnutrition according to GLIM criteria were higher than those without malnutrition, the mean body weight, BMI, MUAC, and calf circumference were lower (Table 1, *p* < 0.05).

**Table 1:** Descriptive characteristics of patients according to the presence of malnutrition based on GLIM criteria

Descriptive characteristics	All groups (*n* = 669)	Malnutrition ( +) (*n* = 147)	Malnutrition ( – ) (*n* = 522)	*p*
	%(*n*) Mean ± SD	%(*n*) Mean ± SD	%(*n*) Mean ± SD	
Age (years)	75.5 ± 7.7	77.0 ± 7.6	75.0 ± 7.7	0.002*
Sex				
Female	55.2 (369)	51.0 (75)	56.3 (294)	0.254**
Marital status				
Married	74.1 (496)	70.1 (103)	75.3 (393)	0.202**
Education level				
Primary school and below	83.1 (556)	84.4 (124)	82.2 (432)	0.648**
Smoking				
Current smoker	6.1 (41)	97.3 (143)	92.9 (485)	0.051**
Alcohol consumption				
Yes	1.0 (7)	0.0 (0)	1.3 (7)	0.158**
Comorbidities				
Yes	60.2 (403)	67.3 (99)	58.2 (304)	0.046**
Surgical operation in the last 3 months				
Yes	20.8 (139)	18.4 (27)	21.5 (112)	0.415**
Food allergy				
Yes	3.3 (22)	4.1 (6)	3.1 (16)	0.542**
Nutritional route				
Oral	95.1 (636)	85.0 (125)	97.9 (511)	< 0.001**
Oral + enteral	2.5 (17)	8.8 (13)	0.8 (4)	
Other^***^	2.4 (16)	6.1 (9)	1.3 (7)	
Disease-specific diet status				
Yes	45.0 (301)	34.0 (50)	48.1 (251)	0.002**
Hospitalization unit				
Internal units	81.5 (545)	85.7 (126)	80.3 (419)	0.133**
Hospitalization(day)	4.2 ± 8.1	5.6 ± 15.0	3.8 ± 4.6	0.005*
Anthropometric measurement				
Weight (kg)	72.9 ± 14.7	65.3 ± 14.5	75.2 ± 14.0	< 0.001
Height (cm)	163.4 ± 8.5	163.3 ± 8.7	163.4 ± 8.5	0.885
BMI (kg/m^2^)	27.3 ± 5.2	24.4 ± 4.7	28.2 ± 5.1	< 0.001
MUAC (cm)	28.5 ± 4.1	26.5 ± 3.9	29.1 ± 3.9	< 0.001
Calf circumference (cm)	33.9 ± 4.6	31.6 ± 4.4	34.5 ± 4.5	< 0.001

*BMI* Body Mass Index. *MUAC* Mid-Upper Arm Circumference

*Mann–Whitney *U* test

**Pearson Chi-Square test (Column percentage)

***Enteral, Parenteral, Enteral + Parenteral

According to GLIM criteria, 22.0% of patients were malnourished. According to MST, MUST, SNAQ, NRS-2002, and GMS, the proportions of patients at risk of malnutrition were 22.9%, 29.6%, 30.2%, 24.8%, and 42.6%, respectively. According to the MNA-SF screening tool, the proportion of patients with malnutrition and at risk of malnutrition was 50.1% (Table 2).

**Table 2:** Prevalence of malnutrition and malnutrition risk according to GLIM Criteria and six different nutritional screening tools

Nutritional screening tools	Malnutrition/Malnutrition Risk	% (*n*)
GLIM criteria	Malnutrition	22.0 (147)
	Non-malnutrition	78.0 (522)
Phenotypic criteria	Involuntary body weight loss	26.8 (179)
	Low BMI	10.0 (67)
	Reduced muscle mass	6.0 (40)
Etiologic criteria	Reduced food intake or malabsorptive disorder	28.1 (188)
	Disease burden/inflammation	21.4 (143)
MST	At risk	29.9 (200)
	No risk	70.1 (469)
MUST	Medium–high risk	29.6 (198)
	Low risk	70.4 (471)
SNAQ	At risk	30.2 (202)
	No risk	69.8 (467)
MNA-SF	Malnutrition + At risk	50.1 (335)
	No risk	49.9 (334)
NRS-2002	At risk	24.8 (166)
	No risk	75.2 (503)
GMS	At risk	42.6 (285)
	No risk	57.4 (384)

*GLIM* Global Leadership Initiative on Malnutrition. *MST* Malnutrition Screening Tool. *MUST* Malnutrition Universal Screening Tool. *SNAQ* Short Nutritional Assessment Questionnaire. *MNA-SF* Mini Nutritional Assessment Short Form. *NRS-2002* Nutritional Risk Screening-2002. *GMS* Graz Malnutrition Screening Tool

**Fig. 2:**
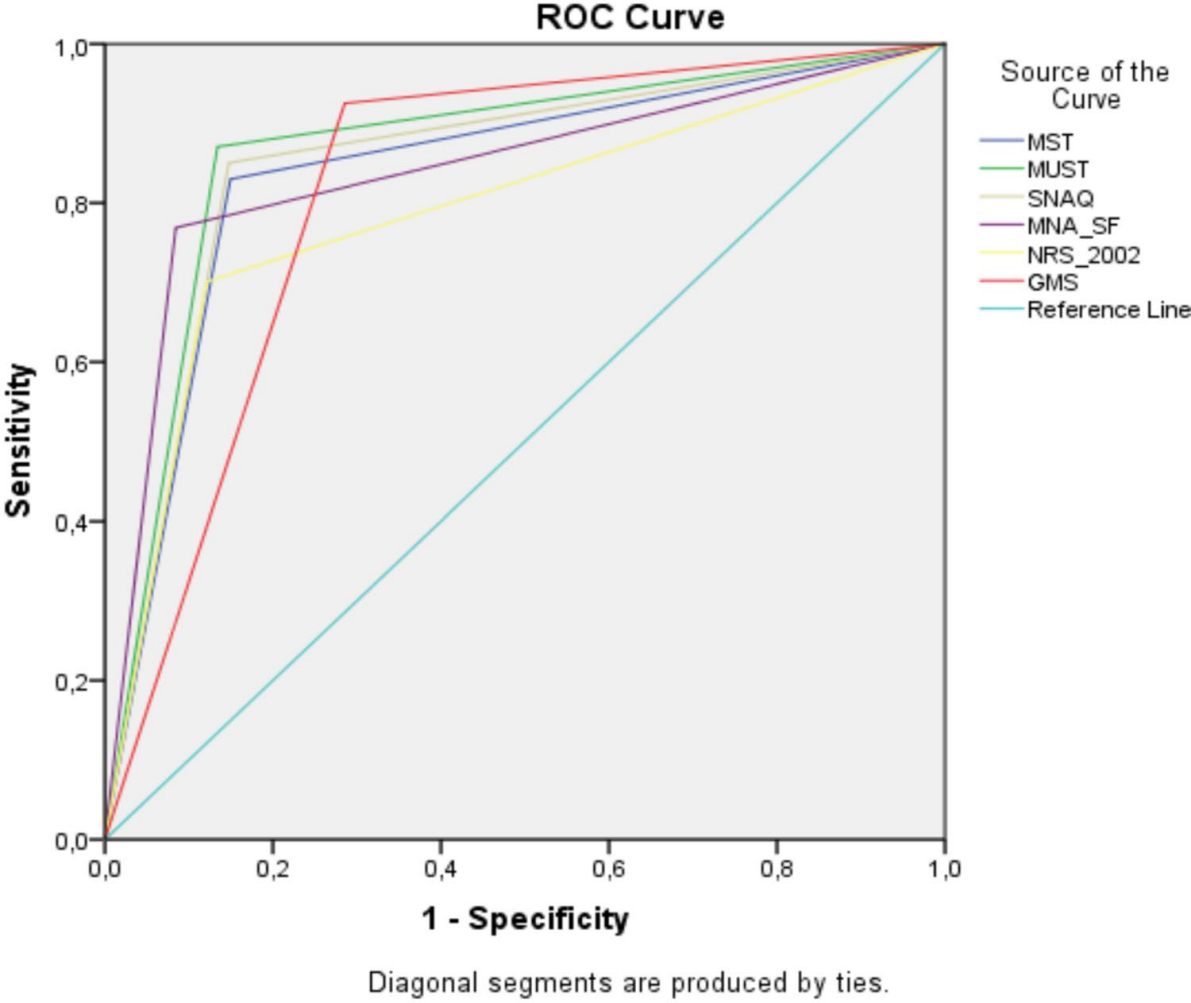
ROC curves of the six nutritional screening tools in determining malnutrition risk

The performance of each nutritional screening tool in detecting the risk of malnutrition according to GLIM criteria is shown in Table 3. The AUC calculated by ROC showed that MST (AUC:0.905) had an excellent predictive value, MUST (AUC:0.874), SNAQ (AUC:0.851), MNA-SF (AUC:0.842) and GMS (AUC:0.820) had good predictive value, while NRS-2002 had a moderate predictive value (Table 3, Fig. 2). In patients, GMS was the most sensitive (92.5%) tool and NRS-2002 was the least sensitive (70.1%) compared with GLIM criteria. MNA-SF had the highest specificity (91.6%), while GMS had the lowest specificity (71.5%). MNA-SF had the highest + LR (9.1), while GMS had the lowest + LR (3.2). NRS-2002 had the highest -LR (0.3), while GMS had the lowest -LR (0.1). MNA-SF had the highest PPV (71.9%), while GMS had the lowest PPV (47.7%). Based on the Cohen κ value, it was found that MUST, SNAQ, and MNA-SF screening tools were moderately compatible with GLIM criteria in detecting the risk of malnutrition, while MST, NRS-2002, and GMS screening tools were in fair agreement with GLIM criteria (Table 3).

**Table 3:** Performance of six nutritional screening tools in detecting the risk of malnutrition according to GLIM criteria

Nutritional screening tools	AUC (95%CI)	Sensitivity (%)	Specificity (%)	LR ( +) (%)	LR ( – ) (%)	PPV (%)	NPV (%)	Accuracy	Cohen κ
MST	0.905(0.880–0.930)*	83.0	85.1	5.6	0.2	61.0	94.7	84.6	60.2*
MUST	0.874(0.838–0.909)*	87.1	86.6	6.5	0.1	64.6	95.9	86.7	65.5*
SNAQ	0.851(0.814–0.889)*	85.0	85.2	5.8	0.2	61.9	95.3	85.2	62.0*
MNA-SF	0.842(0.800–0.885)*	76.9	91.6	9.1	0.3	71.9	93.4	88.3	66.8*
NRS-2002	0.790(0.743–0.837)*	70.1	87.9	5.8	0.3	62.0	91.3	84.0	55.4*
GMS	0.820(0.785–0.855)*	92.5	71.5	3.2	0.1	47.7	97.1	76.1	47.8*

*AUC* Area Under the Curve. *LR (* +*)* Positive Likelihood Ratio. *LR (* – *)* Negative Likelihood Ratio. *PPV* Pozitive Predictive Value. *NPV* Negative Predictive value. *MST* Malnutrition Screening Tool. *MUST* Malnutrition Universal Screening Tool. *SNAQ* Short Nutritional Assessment Questionnaire. *MNA-SF* Mini Nutritional Assessment Short Form. *NRS-2002* Nutritional Risk Screening-2002. *GMS* Graz Malnutrition Screening Tool

*p < 0.001

## Discussion

This study aimed to compare the performance of six different screening tools with GLIM criteria in detecting the risk of malnutrition in hospitalized older adults with cardiovascular disease. Detection of malnutrition status in older adults with cardiovascular disease is very important in terms of improving disease progression and health costs [41]. In the present study, we determined the prevalence of malnutrition in hospitalized older adults with cardiovascular disease and compared the performance of six different screening tools (MUST, SNAQ, MNA-SF, MNA-SF, MST, GMS, NRS-2002), which are frequently used to determine the risk of malnutrition, with GLIM criteria.

In our study, malnutrition was found in 22% of patients according to GLIM criteria. Hirose et al. (2021) reported that 42% of older adults with heart failure were malnourished according to GLIM criteria [42]. Yamaguchi et al. (2023) reported the prevalence of malnutrition according to GLIM criteria as 71% in cardiac patients, 64% in patients with cerebrovascular disease, and 62% in patients with aortic disease [43]. It is thought that the low prevalence of malnutrition in our study according to GLIM criteria may be because approximately 80% of the patients were hospitalized in internal units and had not undergone surgical operation in the last 3 months. In the literature, it is reported that the prevalence of malnutrition is higher in patients who need surgical treatment compared to other patients [44, 45]. In another study conducted by Kootaka et al. (2020) in hospitalized individuals with cardiovascular disease, the prevalence of malnutrition according to GLIM kits was reported as 19%. This prevalence value is lower than the value obtained in our study [19]. This difference is thought to be because the study was conducted with patients aged 20 years and older. The prevalence of malnutrition increases with age. It was reported that the mean age of patients with malnutrition was significantly higher than patients without malnutrition [19]. According to the results obtained using screening tools in our study, the prevalence of malnutrition risk ranged between 24.8% and 50.1%, with the lowest and highest prevalence rates determined by NRS-2002 and MNA-SF, respectively. In our study, the proportion of patients with malnutrition and patients at risk of malnutrition was calculated as 50.1% in the MNA-SF assessment. The prevalence of malnutrition calculated with the other screening tools MST, MUST, SNAQ, and GMS were 29.9%, 29.6%, 30.2%, and 42.6%, respectively. A high prevalence value (42.6%) may have been found in the GMS assessment because it scores the reasons for the decrease in food intake in the last months separately. To detect malnutrition risk and malnutrition quickly and accurately, it is of great importance to choose the screening tool to be used according to the diagnosis of the patient. In a study, patients with heart failure were evaluated with MNA-SF, and malnutrition was detected in approximately 23% of patients [46]. In another study conducted by Jayawardena et al. (2016) with cardiac patients, the prevalence of malnutrition was reported as 22.7%, 40%, 47.9%, and 50.0% with SNAQ, MUST, MST, and NRS, respectively [47]. The fact that the prevalence values reported in the studies in the literature are different from our study may be due to the different age groups and comorbidities of the patients or the different assessment times. On the other hand, the fact that the highest prevalence value was found with MNA-SF [69.6%] in the study by Jayawardena et al. (2016) may be due to the combination of malnutrition risk and malnutrition as in our study [47].

Early detection of the risk of malnutrition using a validated screening tool and better nutritional care in the clinical setting leads to a reduction in the incidence of malnutrition. Malnutrition is undesirable in patients with cardiovascular disease as it is associated with poor functional recovery and adverse clinical outcomes such as mortality, length of hospital stay, and cost of health care. Therefore, early detection of malnutrition with validated malnutrition screening tools is highly recommended [48].

Since the type of disease is an important factor in determining the risk of malnutrition, the diagnosis of the individual should be considered when selecting a screening tool. In clinical practice, the use of screening tools that have been validated for the target population and have good sensitivity and specificity may be useful in accurately detecting malnutrition or the risk of malnutrition. There is no clear information in the literature on which screening tool to use for the detection of malnutrition risk in older adults with cardiovascular disease [18]. On the other hand, there is no study in the literature comparing the nutrition screening tools frequently used in the clinic in older adults with cardiovascular disease. For this reason, we think that the results of our study will make an important contribution to screening processes. According to our results, MST (AUC: 0.905) predicted the risk of malnutrition in hospitalized older adults with cardiovascular disease excellently, while MUST (AUC: 0.874), SNAQ (AUC: 0.851), MNA-SF (AUC: 0.842), GMS (AUC: 0.820) predicted good and NRS-2002 (AUC: 0.790) predicted moderate. Furthermore, GMS had the highest sensitivity and MNA-SF the highest specificity among the tools used to detect malnutrition risk in hospitalized older adults with cardiovascular disease. MNA-SF had the highest agreement with GLIM criteria (Cohen κ: 66.8) and the highest accuracy (88.3%). The fact that MNA-SF has the highest agreement with GLIM criteria and its results are accurate according to GLIM criteria emphasizes its potential importance in detecting malnutrition, especially in the clinical assessment of older adults with cardiovascular disease. Although the MST is recommended by the Norwegian Directorate of Health as a universal screening tool for all adults our results suggest that MNA-SF demonstrates the highest specificity, accuracy, and agreement with GLIM criteria in this population. This highlights the importance of population-specific screening tool selection rather than a one-size-fits-all approach [22].

In a meta-analysis, MNA scores were found to be the strongest predictor of mortality in heart failure compared with other screening tools [49]. The prevalence of heart failure increases with age. It has been stated that the MNA, which is specifically designed for older adults, can be used as the ‘gold standard' to evaluate the validity of different tools in future studies with patients with heart failure until a consensus is reached [50, 51]. Considering that the mean age of the patients in our study was 75.46 ± 7.69 years, our results support the view that the use of MNA-SF will provide more accurate results in patients with cardiovascular disease. In a study, MNA-SF was reported to be the tool with the highest diagnostic ability for assessing the nutritional status of heart failure patients [52]. Compared to the MUST score, the MNA-SF was found to provide a more comprehensive assessment of malnutrition, taking into account weight loss, and the impact of acute illness on food intake, mobility, and neuropsychological problems. Therefore, the MNA-SF has been reported to be the best tool for detecting malnutrition in chronic heart failure compared to MUST and the Subjective Global Assessment (SGA) screening tool [53]. According to our results, MNA-SF was the screening tool that best predicted the risk of malnutrition and our study result is consistent with the studies in the literature.

According to our results, GMS is the screening tool with the highest sensitivity and the best predictor of the prevalence of malnutrition risk. The GMS screening tool has a specificity of 71.5%, which may increase the likelihood of individuals without malnutrition problems being incorrectly referred for further evaluation. Specificity is an important factor to consider, especially in terms of service delivery and staff resources.

In our study, the sensitivity and specificity of GMS, MST, MUST and SNAQ screening tools were found to be above 80.0% concerning GLIM criteria. On the other hand, the specificity of all screening tools except GMS was above 80%. Since the presence of malnutrition, not risk is evaluated with GLIM criteria, it is not a correct approach to compare GLIM criteria and screening tools in terms of specificity and sensitivity [54]. In addition, the NPV values of all screening tools in this study were above 90%. This result can be interpreted as more than 90% of the patients in whom screening tools did not detect a risk of malnutrition were not malnourished.

Our study presents significant results; however, it also has certain limitations. First, the study was conducted in a single center, and the sample size may be limited, particularly for subgroups, which could restrict the generalizability of the results. The collection of some data based on self-reported may have led to reporting bias. Additionally, using the GLIM criteria as a reference inherently incorporates the limitations specific to these criteria. Furthermore, the subjective nature of some screening tools may introduce variability depending on the assessor's experience. Therefore, replicating the study with larger sample groups and data from multiple centers could enhance the generalizability of the results.

## Conclusions

This study evaluated the performance of six different nutritional screening tools (NRS-2002, MST, GMS, MUST, SNAQ, and MNA-SF) in identifying malnutrition risk among older adults with cardiovascular diseases, using GLIM criteria as the reference standard. The results revealed that MNA-SF demonstrated the highest accuracy and agreement with GLIM criteria, emphasizing its potential as a preferred tool in clinical practice for this patient group. MST showed excellent performance in predicting malnutrition risk, while MUST and SNAQ exhibited good predictive capabilities. GMS, although a relatively new screening tool, demonstrated fair agreement with GLIM criteria, contributing to the growing body of evidence on its applicability. The study also highlighted significant variability in the sensitivity and specificity of the screening tools, influenced by factors such as patient demographics, comorbidities, and clinical settings. These results underscore the importance of selecting disease-specific screening tools that align with the unique characteristics of the target population. Overall, this research provides critical insights into the utility of nutritional screening tools in identifying malnutrition risk in older adults with cardiovascular disease. The results can guide healthcare professionals in making informed decisions regarding malnutrition screening and contribute to the development of more targeted and effective nutritional interventions. Further large-scale, multicenter studies are recommended to validate these results and enhance the generalizability of the results.

## Data Availability

The data that support the results of this study are available from the corresponding author, KTS upon reasonable request.
